# Estimating vaccine effectiveness against COVID-19 using cause-specific sick leave as an indicator: a nationwide population-based cohort study, Norway, July 2021 – December 2022

**DOI:** 10.1186/s12889-024-19374-0

**Published:** 2024-07-11

**Authors:** Hinta Meijerink, Lamprini Veneti, Anja Bråthen Kristoffersen, Anders Skyrud Danielsen, Melanie Stecher, Jostein Starrfelt

**Affiliations:** 1 Department of Infection Control and Vaccines, Norwegian Institute of Public Health, Oslo, Norway; 2 Department of Infection Control and Preparedness, Norwegian Institute of Public Health, Oslo, Norway; 3 Department of Method Development and Analytics, Norwegian Institute of Public Health, Oslo, Norway; 4 Department of Microbiology, Oslo University Hospital, Oslo, Norway; 5 Field Epidemiology Path (EPIET), European Centre for Disease Prevention and Control (ECDC), ECDC Fellowship Programme, Stockholm, Sweden; 6 Division for Social Statistics, Statistics Norway, Oslo, Norway

**Keywords:** Norway Real-world evidence, COVID-19, SARS-CoV-2, COVID-19 vaccines, Vaccine effectiveness, Sick leave, Surveillance, Population registers, Cohort studies

## Abstract

**Background:**

Due to changes in testing policy and increased use of rapid tests, other indicators for SARS-CoV-2 infections are needed to monitor vaccine effectiveness (VE). We aimed to estimate VE against COVID-19 sick leave (> 3 days, certified by a medical professional) among employed individuals (25–64-years-old) in Norway.

**Methods:**

We performed a nationwide cohort study by collating data from the Emergency preparedness register for COVID-19. We used adjusted Cox proportional hazard models with vaccine status as a time-varying covariate and presented results as adjusted hazard ratios (aHRs) with corresponding 95% confidence intervals. Separate models were run against sick leave and against SARS-CoV-2 infections during the Delta period (June-December 2021), and against sick leave during the Omicron period (January-December 2022) when SARS-CoV-2 PCR-testing was replaced by rapid self-tests and infections were underreported.

**Results:**

We included 2,236,419 individuals during the Delta period, of whom 73,776 (3.3%) had a reported infection and 54,334 (2.4%) were registered with sick leave. Of the 2,206,952 included individuals in the Omicron period, 300,140 (13.6%) were registered with sick leave. During the Delta period, 55% (26,611) of individuals who had registered sick leave also had a positive test, compared to 32% (96,445) during the Omicron period. The VE against sick leave during the Delta period followed a similar waning pattern to that against SARS-CoV-2 infections. After the second and third dose, the lowest aHRs were estimated for 2–7 days after vaccination for both sick leave (0.25; 95%CI 0.24–0.26 and 0.26; 95% CI 0.24–0.29) and infection ( 0.16; 95% CI 0.15–0.17 and 0.18; 95% CI 0.16–0.19) respectively. During the Omicron period, aHRs for sick leave were higher than during the Delta period, but the lowest aHRs were still found in 2–7 weeks after receiving the second (0.61; 95% CI 0.59–0.64) or third dose (0.63; 95% CI 0.62–0.64).

**Conclusion:**

Our results showed that sick leave could be a relevant indicator for VE in the surveillance of COVID-19 and a finding that may be important in the surveillance of other respiratory infection.

**Supplementary Information:**

The online version contains supplementary material available at 10.1186/s12889-024-19374-0.

## Background

COVID-19 vaccines have shown high efficacy in clinical trials and good effectiveness from observational studies. However, all vaccines have shown waning over time and effectiveness vary against different variants, with lower protection against the Omicron variant compared to Delta [1–9]. Therefore, monitoring of vaccine effectiveness (VE) is important to guide policies and recommendations. VE against COVID-19 has often been measured against either infection (positive polymerase chain reaction (PCR) test), or more severe outcomes such as hospitalisation and death. Using these outcomes can be challenging, for example due to changes in testing policy.

Throughout the pandemic, the Norwegian COVID-19 regulations and measures have changed, including requirements for isolation of SARS-CoV-2 positive individuals, quarantine of close contacts and testing. COVID-19 testing has been recommended for anyone with symptoms, and until September 2021 also required for close contacts. The duration of isolation for COVID-19 cases ranged anywhere from 5 days (after September 2021) to 10 days (before September 2021) and until September 2021 close contacts were required to quarantine themselves. Subsequently, testing and quarantine of close contacts was only recommended (not required) for those unvaccinated. With the introduction of the Omicron variant in November 2021, COVID-19 measures were reinforced, including a seven-day isolation of cases, testing and quarantine of household members. By mid-February 2022, all isolation requirements were lifted, with a four-day isolation recommended for positive cases (not required) [10].

In Norway, SARS-CoV-2 PCR-testing has been free and widely available since summer 2020 and by the end of 2021 self-administered rapid testing was freely available. The reporting of any positive PCR-tests to the Norwegian Surveillance System for Communicable Diseases (MSIS) by both laboratories and clinicians is mandatory by law, but rapid tests are not included in the surveillance [11]. Until mid-January 2022, positive rapid tests needed to be confirmed with a PCR-test and were thus registered in MSIS. Due to a policy change not requiring this confirmation as well as lower severity of the circulating Omicron variant [12], the number of SARS-CoV-2 infections are known to be underreported after January 2022. After this, COVID-19-related hospitalisations and deaths became even more important surveillance indicators to monitor COVID-19 as well as the impact of vaccination programme. However, COVID-19 symptomatic cases also contribute to the burden on society, especially those requiring a physician-certified medical leave (hereafter “COVID-19 sick leave”). In Norway, all employees have the right to paid sick leave up to three consecutive days without needing a medical certification and a majority of employees are covered by collective labour agreements that entitle them to more days than this. When the length of an employee's sick leave crosses the threshold set in their agreement, they will need a physician to issue a certified medical leave to receive pay [13], and this is registered in the Norwegian Registry for Primary Health Care (NRPC). In addition, sick leave may also be provided when mandatory isolation or quarantine is required. Leave to take care for a child is mandated by law in Norway and does not require a medically certified sick leave [13, 14]. Therefore, sick leave could be a useful indicator of the burden of COVID-19, reflecting symptomatic disease among the working population that would not otherwise be registered as it does not necessitate hospitalisation, an aspect important for health economics and health technology assessment. In this project we aimed to assess vaccine effectiveness by estimating the adjusted hazard ratios (aHRs) for vaccination against COVID-19 sick leave among employed individuals (25–64-years-old) in Norway from July 2021 to December 2022.

## Methods

### Study population

**Fig. 1:**
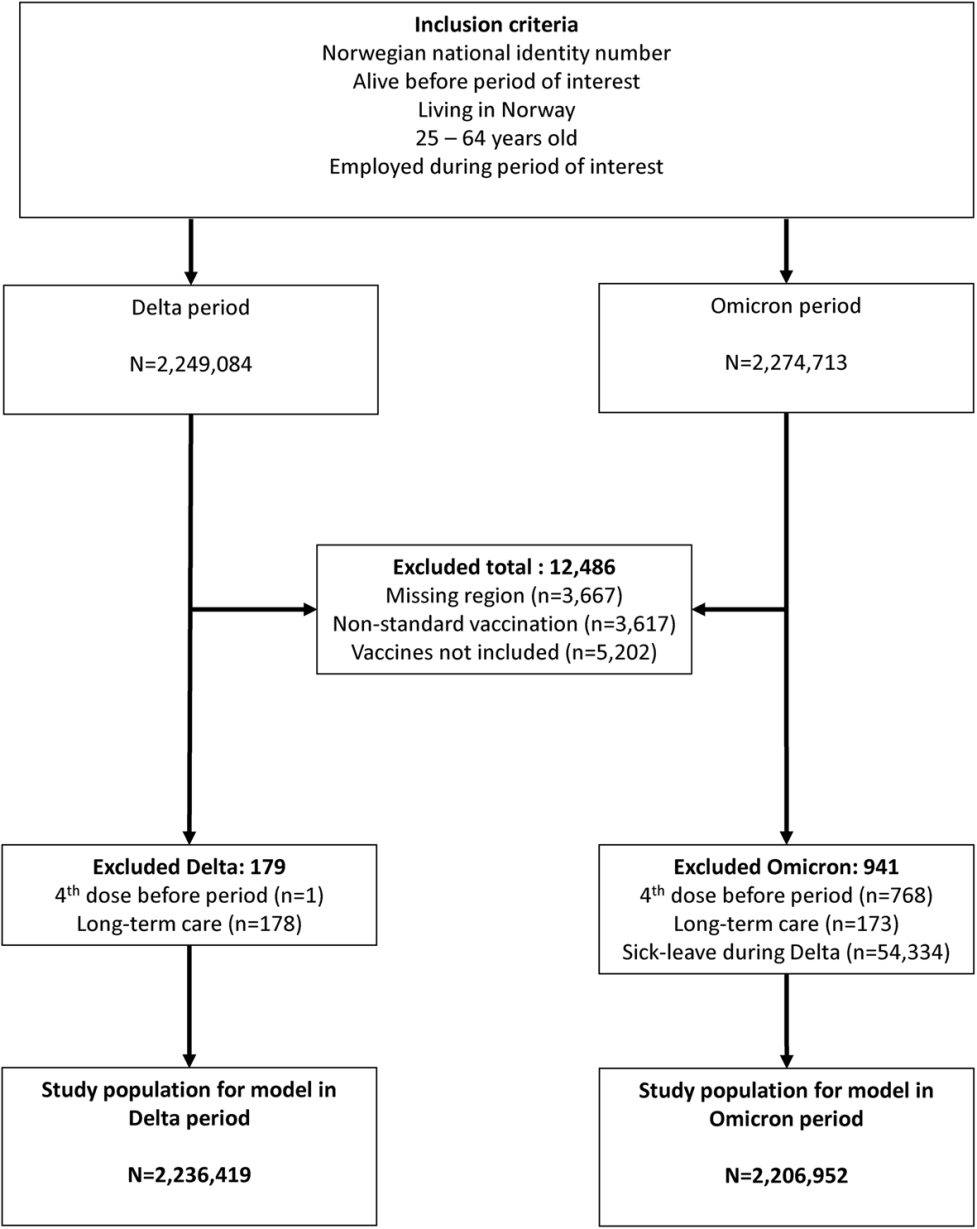
Flowchart showing the selection of study populations and reasons for exclusion from analyses. Delta period from 19 July – 19 December 2021, Omicron period from 3 January – 31 December 2022

For this nationwide cohort study, we collated data from the Emergency preparedness register for COVID-19 (Beredt C19) (Additional file, table S1), which contains individual-level data [15]. We included employed individuals, aged 25–64 years with a valid Norwegian national identity number registered as living in Norway. Individual-level data used for this study included data regarding COVID-19 vaccination (type and dates), COVID-19 specific sick leave dates, age, sex, county of residence, risk groups based on underlying comorbidities, crowded living conditions, and testing dates for COVID-19 positive PCR tests. On 31 March 2023, we extracted data for the period from 19 July 2021 to 31 December 2022. See additional file for further details on data sources. Figure 1 gives an overview of the exclusion criteria applied to the study population; individuals were included if they were employed at any time during the study period.

### Definitions

The primary outcomes for this study were SARS-CoV-2 infection and COVID-19 sick leave. The following definitions were used to define these outcomes:

*SARS-CoV-2 infection****:*** a positive SARS-CoV-2 PCR test reported to the MSIS register. We used testing date as time of infection (positive PCR test) and included only the first SARS-CoV-2-infection per individual to reduce biases related to natural immunization. Both symptomatic and asymptomatic reported cases have been included as it is not possible to distinguish between these in MSIS.

*COVID-19 sick-leave:* disease requiring a physician certified medical leave where COVID-19 was set as the primary diagnosis by a primary health provider. These are needed when the length of sick leave is more than three consecutive days and when it crosses the threshold set in employees’ agreements.

The main variable of interest is COVID-19 vaccine status, which is included in all models as time-varying exposure. COVID-19 vaccine status was defined based on time since receiving the last vaccine dose based on dates reported in the Norwegian Immunisation Registry (SYSVAK):

- Unvaccinated: unvaccinated, used as reference level in Cox regressions.
- 1st dose: ≥ 21 days after first vaccine dose.
- 2nd dose: ≥ 7 days after the 2nd dose up to 3rd dose, divided in period of six weeks.
- 3rd dose: ≥ 7 days after the 3rd dose, divided in period of six weeks.

Adjustment variables in the model include:

*Age*: based on year of birth.

*Sex*: “Male” or “Female”.

*Region of residence*: six regions based on NUTS2 code.

*Country of birth*: three levels: “born in Norway”, “born outside of Norway” and “unknown”. Country of birth was considered a confounder as there is a demonstrated difference in vaccine uptake as well as in infection rates [16].

*Risk group*: three levels based on pre-existing medical conditions; “low”, “medium” and “high”.

The additional file includes detailed information on the variables included in the models.

### Data analyses

We used Cox proportional hazard models to estimate the aHRs with corresponding 95% confidence intervals (95%CI) associated with different vaccine statuses against SARS-CoV-2 infection and COVID-19 sick leave. Vaccine effectiveness was calculated as (1-aHR)*100 and reported in the supplementary file.

We performed the analyses for two separate time periods based on at least 80% of the sampled sequences being the specific variant: Delta period from 19 July to 19 December 2021 and Omicron period from 3 January to 31 December 2022. As SARS-CoV-2 positive PCR-test was not a reliable indicator during the Omicron period, we only included SARS-CoV-2 infection as outcome for the Delta period whereas COVID-19 sick leave was run for both periods.

All models used a calendar time scale and vaccine status was included as a time-varying exposure and allowed for different baseline hazards for other covariates by stratifying using the function strata of the *survival* package (version 3.1.12) in RStudio (R-version 4.0.2) [17, 18]. Unvaccinated individuals were used as reference and the aHR was reported for all levels of the vaccine status factor with more than five events. We adjusted all models for 10-year age bands, sex region of residence, country of birth, and risk group. We stopped follow-up time at the time of an event (SARS-CoV-2 infection or sick leave respectively), time of death, or end of variant specific follow-up period. We also right censored individuals at time of hospitalisation when no sick leave was reported, as we only have access to sick leave reported by primary health care.

## Results

### Study population selection and characteristics

Table 1 shows the characteristics and total number of events among the study population during the Delta and Omicron periods included in this study. We included 2,236,419 individuals during the Delta period, of whom 73,776 (3.3%) had a reported infections and 54,334 (2.4%) required COVID-19 specific sick leave. During the Omicron period, we included 2,206,952 individuals, of whom 300,140 (13.6%) required COVID-19 specific sick leave. During the Delta period, 26,611 (55%) of the 54,334 individuals who had reported sick leave had a positive test, compared to 96,445 (32%) of the 300,140 during the Omicron period.

**Table 1:** Study population characteristics (25–64-years-old employed individuals) during the Delta and Omicron periods in Norway

		**Total study population, n (%)**	**Infections, n (%)**	**Sick Leave, n (%)**
**Delta period** *(19.07–19.12.2021)*		**2 236 419**	**73 776**	**54 334**
Age group	25–34	582 228 (26%)	19 794 (26.8%)	16 690 (30.7%)
	35–44	575 499 (25.7%)	23 534 (31.9%)	17 645 (32.5%)
	45–54	588 970 (26.3%)	19 910 (27%)	13 169 (24.2%)
	55–64	489 722 (21.9%)	10 538 (14.3%)	6 830 (12.6%)
Sex	Male	1 158 469 (51.8%)	38 441 (52.1%)	25 277 (46.5%)
	Female	1 077 950 (48.2%)	35 335 (47.9%)	29 057 (53.5%)
Underlying risk	None	1 968 322 (88%)	65 492 (88.8%)	48 021 (88.4%)
	Low-medium	245 899 (11%)	7 682 (10.4%)	5 961 (11%)
	High	22 198 (1%)	602 (0.8%)	352 (0.6%)
Country of birth	Norway	1 739 149 (77.8%)	48 765 (66.1%)	34 352 (63.2%)
	Not Norway	453 465 (20.3%)	24 230 (32.8%)	19 506 (35.9%)
	Unknown	43 805 (2%)	781 (1.1%)	476 (0.9%)
**Omicron period** *(03.01–31.12.2022)*		**2 206 952**		**300 140**
Age group	25–34	568 135 (25.7%)	n.a	84 615 (28.5%)
	35–44	568 607 (25.8%)	n.a	87 311 (29.4%)
	45–54	584 255 (26.5%)	n.a	74 684 (25.1%)
	55–64	485 955 (22%)	n.a	50 530 (17%)
Sex	Male	1 147 964 (52%)	n.a	123 026 (41.4%)
	Female	1 058 988 (48%)	n.a	174 114 (58.6%)
Underlying conditions	None	1 942 684 (88%)	n.a	258 684 (87.1%)
	Low-medium	242 865 (11%)	n.a	36 292 (12.2%)
	High	21 403 (1%)	n.a	2 164 (0.7%)
Country of birth	Norway	1 716 470 (77.8%)	n.a	219 260 (73.8%)
	Not Norway	446 983 (20.3%)	n.a	73 753 (24.8%)
	Unknown	43 499 (2%)	n.a	4 127 (1.4%)

### Vaccine effectiveness

**Fig. 2:**
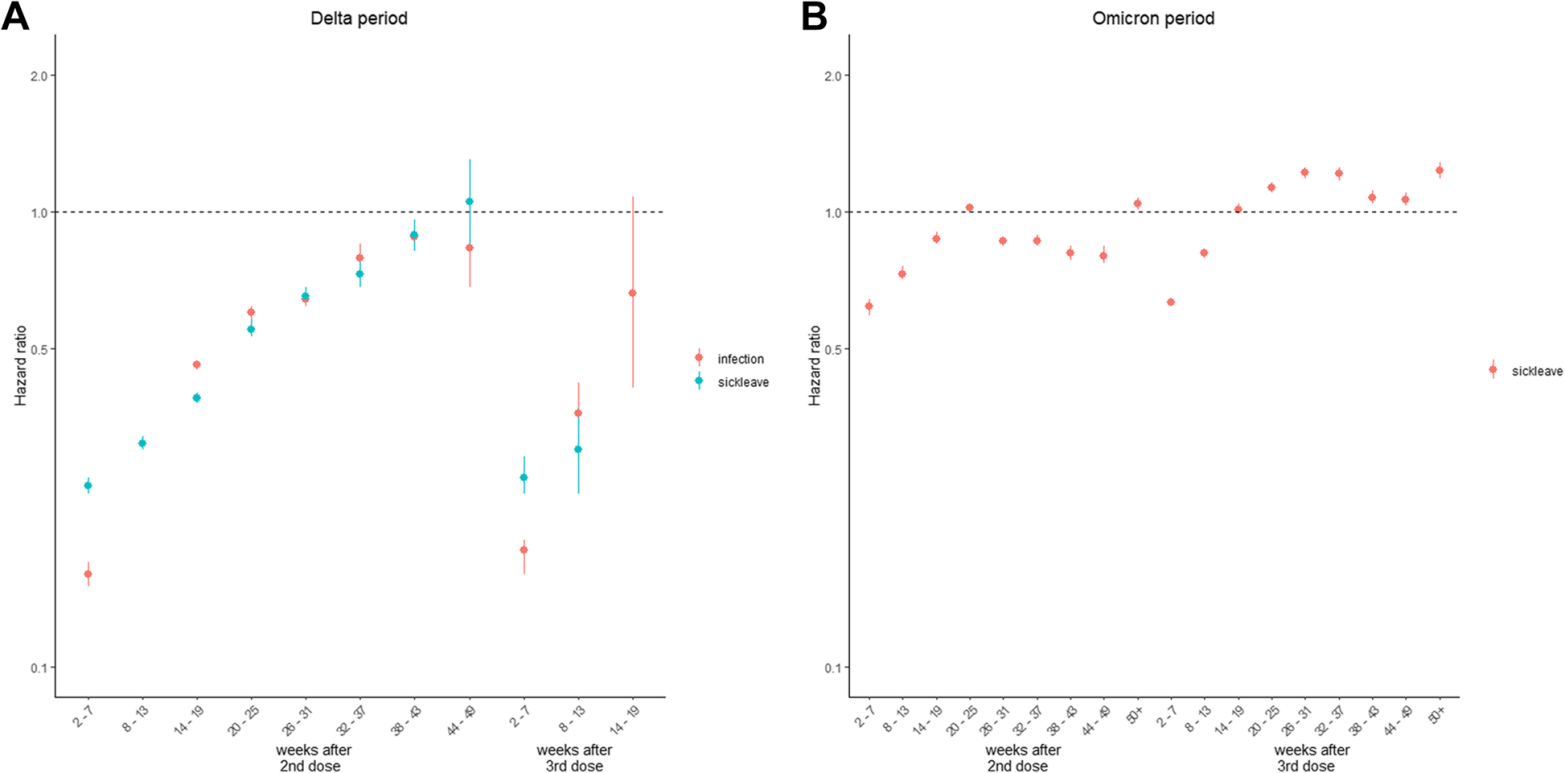
Estimated adjusted hazards ratios against SARS-CoV-2 infection (red) and COVID-19 sick leave (green) among employed individuals (25–64 years) during Delta (**A**) and Omicron (**B**) periods in Norway

During the Delta period, the highest protection was observed in the period right after receiving a vaccine (2–7 weeks), with similar patterns of aHRs for infection and sick leave (Fig. 2A). Compared to unvaccinated individuals, the aHRs for SARS-CoV-2 infection increased from 0.16 (95%CI: 0.15–0.17) 2–7 weeks after receiving the second dose to 0.83 (95%CI: 0.68–1.00) after 44–49 weeks, and from 0.18 (0.16–0.19) 2–7 weeks after receiving the third dose to 0.66 (0.40–1.07) 14–19 weeks after. Similarly, against COVID-19 sick leave the aHR went from 0.25 (0.24–0.26) to 1.05 (0.84–1.30) in the same periods after the second dose (Fig. 2A).

As described above, as SARS-CoV-2 positive PCR-test was not a reliable indicator during the Omicron period, we only included COVID-19 sick leave in these analyses. All estimated aHRs for COVID-19 sick leave after vaccination were closer to 1 during the Omicron period than Delta period (Fig. 1) and thus indicate less protection against sick leave during this period. Similar to the results from the Delta period, the lowest aHRs for sick leave, and this highest protection, were found in the first period (2–7 weeks) after the second (0.62; 0.59–0.64) and third dose (0.63; 0.62–0.64) (Fig. 2B). The aHRs estimates presented in Fig. 1 as well as VE estimates can be found in the additional file (tables S2,S3 and figure S1).

## Discussion

We showed that in a period with free widely available PCR testing against SARS-CoV-2 (Delta period) the estimated aHRs for COVID-19 sick leave indicated a similar protection level and waning pattern as against SARS-CoV-2 infection. In the Omicron period, the protection against COVID-19 sick leave was lower than during the Delta period and highest in the first weeks (2–7 weeks) after receiving a vaccine dose. COVID-19 sick leave may prove a useful indicator to include for COVID-19 surveillance and COVID-19 disease burden estimations, especially in periods with low or absent testing for (mild or less severe) symptomatic disease. As such disease does not require hospitalisation, it would not be registered in other manners. This indicator could also prove important for health economical evaluations and health technology assessment.

The unique Nordic register system allowed both access to individual level information regarding COVID-19 sick leave as well as linking this information with other characteristics. With reduced testing worldwide, countries with access to similar data in available and well-established registries can benefit by using already registered data to estimate the burden of COVID-19 and VE. Since changing the testing recommendation for SARS-CoV-2 in January 2022, we expect that the data on certified COVID-19 sick leave in registers is more complete than those of SARS-CoV-2 infection as the sick leave is a requirement both for the individual to receive paid leave as well as for the GP to receive payment. As such, COVID-19 sick leave will reflect the burden of COVID-19 resulting in symptomatic disease for a prolonged period of time.

The results from this study show consistent patterns with VE estimates against other outcomes in literature, including higher VE against Delta than Omicron, and waning with time since last dose [1–9]. There are periods in which we estimate an aHR for sick leave of more than 1 in the vaccinated, which may indicate residual confounding. This residual confounding may be due to behavioural differences between those unvaccinated and the rest of the population. Such behavioural aspects are difficult to capture as covariates in a regression model, especially when using register data. In addition, natural immunity or prioritisation of vaccines to those at highest risk of disease may also play a role. As the unvaccinated group has become smaller and increasingly diverged from the general population, it should be considered to use other estimation processes to investigate patterns of renewed protection and waning due to vaccination [19]. We showed highly congruent results for sick leave and infections during the Delta period and therefore sick leave could be a relevant indicator for surveillance of COVID-19 VE to supplement information gathered from VE estimates against other indicators such as infection and hospital admissions. The registration of sick leave in the Norwegian registers are fully automated and data is thus complete as it is not possible for a physician to certify a medical leave without it being registered. Sick leave may be an especially important indicator, in age groups with low risk of severe outcomes such as hospital admission and when other indicators for (symptomatic) disease are not available or known to be severely under-reported and for economic evaluations.

In addition, using clinical outcomes, such as sick leave and hospitalisation, may be more relevant to guide public health measures targeted to limit the burden on health care systems. Another large advantage of using nation-wide registries is the size of the population included, resulting for high power as well as the ability to do analyses in sub-populations without requiring an effort from health care personnel.

Using COVID-19 specific sick leave has some caveats that could affect the estimations. In the data, we have no information of the duration of sick leave nor whether the sick leave was prescribed for acute or post-acute sequelae of COVID-19. For this last point, we only included the first sick leave reported, but this might still bias our results. A previous study among healthcare workers showed reduced periods of sick leave after a SARS-CoV-2 infection among those previously vaccinated compared to unvaccinated [20]. Since employees in Norway can take a few days of paid sick leave without a medical certificate, this could affect our results. As mentioned in the background, during certain periods of the pandemic cases needed to isolate and close contacts were required to self-quarantine. However, from mid-February isolation of cases was not mandatory and therefore the sick leave reported in the Omicron period are more likely to be symptomatic cases. Even though this may dilute the results, it does still present a burden by the pandemic. Some individuals may not need a medical certified sick leave, even though they are ill for longer than a few days, reasons could be that they are covered by agreements that entitle them to longer period of absence without doctors certification and/or have the ability to work from home [13]. However, there is no reason to believe that this would be associated with vaccine uptake and the ability to work home. There is also no need for a registered sick leave, medically certified or otherwise, if you do not need a leave of absence with pay from a job. This means sick leave cannot be used as an indicator for vaccine effectiveness among students, children and youth still in school, unemployed individuals, or individuals on benefits [13]. We included individuals during the whole period of interested if they were registered as employed at any point during this period. Thus, some individuals may not have worked during the complete period and as such would not need certified sick leave. However, we expect this to have limited impact as this would be a relatively small group and we have no reason to suspect this to be associated with vaccine uptake. In this study, the reference group was unvaccinated individuals. However, in Norway only a small proportion of individuals did not receive any COVID-19 vaccine dose and may therefore be a group with very different characteristics. Testing among vaccinated may differ from unvaccinated individuals, which could affect COVID-19 VE estimates during the Delta period. In addition, we expect that a proportion of those unvaccinated will have natural immunity due to prior infection, therefore the estimated aHRs and VE should not be interpreted as vaccine efficacy. During both periods we included only the first reported event to reduce biases related to immunization through natural exposure. However, we could not account for prior infections in our models, due to changes in testing policies as especially during Omicron. Our estimates may be biased by significant increase of reinfections during the Omicron period [21], due to immune escape of the omicron variant and waning immunity, especially since reinfections may occur more frequently among unvaccinated individuals, which could lead to an underestimation of VE [22].

## Conclusions

In conclusion, access to data on disease specific sick leaves provided a unique ability estimate vaccine effectiveness against an additional relevant outcome to monitor COVID-19 vaccine effectiveness. It is likely that our results can also be applied to other viral, respiratory diseases and influenza-like illness. Therefore, as a preparedness measure, infrastructure should be established so that this data can be incorporated in surveillance programmes. Depending on the timeliness, our findings could be of importance in the ongoing COVID-19 surveillance of the vaccine programme, not just as an indicator of vaccine effectiveness against infection, but also for other studies looking into disease burden or for health economical evaluations.

### Supplementary Information

Supplementary Material 1.

## Data Availability

The datasets analysed for this study come from the national emergency preparedness registry for COVID-19 (Beredt C19), housed at the Norwegian Institute of Public Health. This registry comprises data from a variety of national registries and legal restrictions prevent the researchers from sharing the dataset used in the study. However, external researchers can request access to linked data from the same registries from outside the structure of Beredt C19, as per normal procedure for conducting health research on registry data in Norway (https://www.helsedata.no). Further information on the preparedness registry, including access to data from each data source, is available at: https://www.fhi.no/en/id/infectiousdiseases/coronavirus/emergency-preparedness-register-for-covid-19.

## Abbreviations

95%CI: 95% Confidence intervals
aHRs: Adjusted hazard ratios
COVID-19: Coronavirus 2019
DPIA: Data protection impact assessment
SYSVAK: Norwegian immunisation registry
Beredt C19: Norwegian national preparedness register for COVID-19
MSIS: Norwegian surveillance system for communicable diseases
COVID-19 sick leave: Physician certified medical leave where COVID-19 was set as the primary diagnosis by a primary health provider
PCR: Polymerase chain reaction
SARS-CoV-2: Severe acute respiratory syndrome coronavirus 2
VE: Vaccine effectiveness
NRPC: Norwegian Registry for Primary Health Care
