# Supplementary material for: Estimating vaccine effectiveness against COVID-19 using cause-specific sick leave as an indicator: a nationwide population-based cohort study, Norway, July 2021 – December 2022
Source: BMC Public Health. 2024 Jul 11;24:1861. doi: 10.1186/s12889-024-19374-0 (PMC11241785; doi:10.1186/s12889-024-19374-0)
Supplement: Supplementary file 1 — Supplementary Material 1. [file 12889_2024_19374_MOESM1_ESM.pdf]

Additional file 1

Supplementary data to the manuscript "Estimating vaccine effectiveness against COVID-19 using cause-specific sick leave as an indicator: A nationwide population-based cohort study, Norway, July 2021 – December 2022"

Hinta Meijerink, Lamprini Veneti, Anja Bråthen Kristoffersen, Anders Skyrud Danielsen, Melanie Stecher, Jostein Starrfelt

Table of content

1. DATA SOURCES 2

Table S1. Data sources in the Norwegian preparedness registry (BeredtC19) used in this study and variables retrieved from each source 2

2. DEFINITIONS 3

3. VACCINE EFFECTIVENESS ESTIMATES 5

Table S2. Estimated adjusted hazard ratios and vaccine effectiveness for COVID-19 vaccination status against infection and COVID-19 sick leave during the period of Delta dominance among employed individuals (24-65 years) in Norway, 19 July to 19 December 2021 5

Table S3. Estimated adjusted hazard ratios and vaccine effectiveness for COVID-19 vaccination status against infection and COVID-19 sick leave during the period of Omicron dominance among employed individuals (24-65 years) in Norway, 3 January to 31 December 2022 6

Figure S1. Estimated adjusted vaccine effectiveness for COVID-19 vaccination status against infection and COVID-19 sick leave during the period of Delta (A) and Omicron dominance (B) among employed individuals (24-65 years) in Norway. 7

4. REFERENCES 7

Data sources

All data in this study came from the national emergency preparedness register, Beredt C19 (1). Beredt C19 contains individual-level data from central health registries, national clinical registries, and other national administrative registries. The data sources and variables used are shown in Table S1

Table S1. *Data sources in the Norwegian preparedness registry (BeredtC19) used in this study and variables retrieved from each source.*

**Norwegian Abbreviation**	**Full name of data source**	**Information obtained**
Folkeregisteret	National Population Register	Age, Sex, County of residence*, Country of birth, Date of death
SYSVAK	The National Immunisation Register	Date of vaccination, Vaccine product type
NIPaR	Norwegian Intensive Care and Pandemic Register	Date of hospitalisation, COVID-19 as main cause of admission, Date of ICU admission
MSIS	The Norwegian Surveillance System for Communicable Diseases	Date of sample of SARS-CoV-2 positive test, Date of COVID-19 associated death
MSIS lab database	Table prepared in BeredtC19	Define virus variant for cases that have been screened successfully
NRPC	Norwegian Registry for Primary Health Care	Information on COVID-19 sick leave
Beredt C19 risikogrupper	Table prepared in BeredtC19 Source: Norwegian Patient Registry (NPR): individual level data from all public specialist health-care services in Norway.	Defines risk groups (see definition below)
Aa-registeret	The Register of Employers and Employees	Provides data on employment and time of employment

** The county of residence was updated in January 2022, which might lead to some errors for individuals who have moved the last half year*

Definitions

SARS-CoV-2 infection: We defined SARS-CoV-2 infection as a positive SARS-CoV-2 PCR test reported to the Norwegian Surveillance System for Communicable Diseases (MSIS) registry. We use testing date as time of infection (positive PCR test) and included only the first SARS-CoV-2-infection per individual, to reduce biases related to natural immunization. Both symptomatic and asymptomatic reported cases have been included as it is not possible to distinguish between these in MSIS.

COVID-19 sick leave: illness requiring a physician certified medical leave where COVID-19 was set as the diagnosis by a primary health provider. These are typically needed when the duration of illness is more than three consecutive days.

Variant waves: In Norway, SARS-CoV-2 Delta or Omicron variants were identified using Sanger partial S-gene sequencing, or PCR screening targeting specific single nucleotide polymorphisms, insertions or deletions; details for laboratory testing for variants previously described (2). We defined the Delta dominant wave between 19 July 2021 to 19 December 2021 and the Omicron dominant wave between 3 January to 30 September 2022; these periods are based on 80% of screened samples belonging to Delta or Omicron respectively.

Country of birth: Country of birth was considered a confounder as there is a demonstrated difference in vaccine uptake as well as in infection rates (3). In this study the variable was based on information in the National population register and divided in "born in Norway", "born outside Norway" or "unknown".

Risk groups: Some underlying medical conditions increase the risk of severe COVID-19 outcomes, regardless of age (source: Beredt C19 risikogrupper). These individuals have been prioritised in the vaccination campaigns in Norway. This data is last updated late 2021 which means all newly diagnosed cases are not identified. However, the classification is highly predictive of chance of severe covid disease. The underlying comorbidities that have been defined as increasing the risk of severe COVID-19 are divided into two groups:

High risk: people with diseases/conditions that carry a high risk of severe COVID-19:

- Organ transplant
- Immunodeficiency
- Haematological cancer in the last five years
- Other active cancers
- Neurological or neuromuscular diseases that cause impaired cough or lung function (e.g., ALS and cerebral palsy)
- Chronic kidney disease, or significant renal impairment.

Low-Medium risk: people with diseases/conditions that entail a moderate risk of severe COVID-19:

- Chronic liver disease or significant hepatic impairment
- Diseases requiring immunosuppressive therapy
- Diabetes
- Chronic lung disease including cystic fibrosis and severe asthma which have required the use of high dose inhaled or oral steroids within the past year
- Obesity with a body mass index (BMI) of ≥35 kg/m2

- Dementia
- Chronic heart and vascular disease (with the exception of high blood pressure) and stroke

Employment: Individuals registered as being employed in the State Register of Employers and Employees (AA-registeret). Employment was considered independently in the two periods, i.e. individuals could be considered employed during Delta, Omicron or both.

Vaccine status: vaccine status is used as a time-varying variable defined on the date and number of doses received (SYSVAK) during the study period. The first seven days after 2nd or 3rd dose were included in the models as a separate factor level but not reported in this manuscript, similarly for the first 21 days after the 1st dose. Since July 2022 until now, the 4th dose is only recommended for elderly and risk groups and thus excluded from our analyses. For the models we used the following grouping for vaccine status:

- Unvaccinated: unvaccinated up to seven days before the first dose, used as reference level in Cox regressions.
- 1st dose: ≥21 days after first vaccine dose up to seven days after second vaccine dose
- 2nd dose: >7 days after the 2nd dose, divided in period of six weeks
- 3rd dose: >7 days after the 3rd dose, divided in period of six weeks

Vaccine effectiveness estimates

Tables S2 and S3 show the estimated hazard ratios for during the Delta and Omicron periods, respectively.

Table S2. *Estimated adjusted hazard ratios and vaccine effectiveness for COVID-19 vaccination status against infection and COVID-19 sick leave during the period of Delta dominance among employed individuals (24-65 years) in Norway, 19 July to 19 December 2021*

Vaccination status		Person years	Events (n)	Hazard ratio (95% CI)*		Vaccine effectiveness (95%CI)*#	
**SARS-CoV-2 infection**							
Unvaccinated		90 638	14 978	*reference*		*reference*	
1st dose	<21 days	44 622	1 242	0.43	(0.40 - 0.45)	57.3	(54.6 - 59.9)
	≥ 21 days	120 661	5 173	0.46	(0.44 - 0.47)	54.2	(52.7 - 55.8)
2nd dose	<7 days	28 035	918	0.37	(0.35 - 0.40)	62.5	(59.8 - 65.1)
	2 - 7 weeks	178 896	2 380	0.16	(0.15 - 0.17)	83.9	(83.2 -84.7)
	8 - 13 weeks	205 108	9 604	0.31	(0.30 - 0.32)	69.3	(68.5 - 70.2)
	14 - 19 weeks	150 511	22 481	0.46	(0.45 - 0.47)	54.0	(52.9 - 55.0)
	20 - 25 weeks	57 664	7 361	0.60	(0.58 - 0.62)	40.1	(38.3 - 41.9)
	26 - 31 weeks	28 039	6 065	0.64	(0.62 - 0.67)	35.6	(33.4 - 37.6)
	32 - 37 weeks	7 300	802	0.79	(0.74 - 0.85)	20.6	(14.6 - 26.1)
	38 - 43 weeks	3 984	1 034	0.88	(0.82 - 0.94)	12.1	(6.2 - 17.5)
	44 - 49 weeks	217	105	0.83	(0.68 - 1.00)	17.2	(-0.4 - 31.7)
3rd dose	<7 days	5 134	948	0.42	(0.40 - 0.45)	57.6	(54.7 - 60.4)
	2 - 7 weeks	6 955	500	0.18	(0.16 - 0.19)	82.3	(80.6 - 83.8)
	8 - 13 weeks	1 209	165	0.36	(0.31 - 0.42)	64.2	(58.1 - 69.3)
	14 - 19 weeks	74	16	0.66	(0.41 - 1.08)	33.7	(-8.4 - 59.5)
	20 - 25 weeks	20	<5	n.a.		n.a.	
**Sick leave**							
Unvaccinated		90 690	12 386	*reference*		*reference*	
1st dose	<21 days	44 567	1 618	0.48	(0.46 - 0.51)	51.8	(49.1 - 54.4)
	≥ 21 days	120 754	6 040	0.54	(0.52 - 0.56)	46.0	(44.2 - 47.8)
2nd dose	<7 days	27 980	872	0.29	(0.27 - 0.32)	70.6	(68.5 - 72.6)
	2 - 7 weeks	178 453	4 483	0.25	(0.24 - 0.26)	74.6	(73.7 - 75.6)
	8 - 13 weeks	204 646	7 043	0.31	(0.30 - 0.32)	69.0	(68.0 - 70.0)
	14 - 19 weeks	150 699	11 395	0.39	(0.38 - 0.40)	61.1	(60.0 - 62.2)
	20 - 25 weeks	57 687	4 452	0.55	(0.53 - 0.58)	44.6	(42.5 - 46.6)
	26 - 31 weeks	28 161	3 881	0.65	(0.63 - 0.68)	34.9	(32.2 - 37.4)
	32 - 37 weeks	7 298	616	0.73	(0.68 - 0.80)	26.6	(20.3 - 32.4)
	38 - 43 weeks	4 010	670	0.89	(0.82 - 0.96)	11.2	(3.8 - 18.0)
	44 - 49 weeks	229	80	1.05	(0.84 - 1.30)	-4.6	(-30.4 - 16.2)
3rd dose	<7 days	5 110	294	0.24	(0.21 - 0.26)	76.5	(73.6 - 79.1)
	2 - 7 weeks	6 937	422	0.26	(0.24 - 0.29)	73.8	(71.0 - 76.3)
	8 - 13 weeks	1 212	80	0.30	(0.24 - 0.37)	70.1	(62.6 - 76.1)
	14 - 19 weeks	75	<5	n.a.		n.a.	

*Result not shown for strata with <5 events. *Adjusted for age, sex, region of residence, country of birth and risk group. ¤ Vaccine effectiveness= (1-aHR)*100*

Table S3. *Estimated adjusted hazard ratios and vaccine effectiveness for COVID-19 vaccination status against infection and COVID-19 sick leave during the period of Omicron dominance among employed individuals (24-65 years) in Norway, 3 January to 31 December 2022*

**Vaccination status**		**Person years**	**Events (n)**	**Hazard ratio (95% CI)***		**Vaccine effectiveness (95%CI)*#**	
**Sick leave**							
Unvaccinated		133 401	25005	*reference*		*reference*	
1st dose	<21 days	931	355	0.74	(0.67 - 0.82)	25.9	(17.7 - 33.3)
	≥ 21 days	36 827	7293	0.97	(0.94 - 0.99)	3.2	(0.61 - 5.7)
2nd dose	<7 days	820	236	0.59	(0.52 - 0.67)	41.2	(33.2 - 48.3)
	2 - 7 weeks	7 088	2450	0.62	(0.59 - 0.64)	38.4	(35.8 - 40.9)
	8 - 13 weeks	10 646	3422	0.73	(0.71 - 0.76)	26.7	(24.0 - 29.3)
	14 - 19 weeks	34 732	11004	0.87	(0.85 - 0.90)	12.6	(10.5 - 14.7)
	20 - 25 weeks	75 621	37088	1.02	(1.01 - 1.04)	-2.3	(-4.0 - -0.5)
	26 - 31 weeks	53 848	15335	0.86	(0.84 - 0.88)	14.0	(12.2 - 15.8)
	32 - 37 weeks	54 284	5210	0.86	(0.84 - 0.89)	13.8	(11.1 - 16.4)
	38 - 43 weeks	52 234	3964	0.81	(0.78 - 0.84)	19.1	(16.3 - 21.9)
	44 - 49 weeks	50 528	3275	0.80	(0.77 - 0.84)	19.7	(16.4 - 22.8)
	≥50 weeks	154 011	10305	1.04	(1.01 - 1.07)	-3.6	(-6.7 - -0.56)
3rd dose	<7 days	19 076	4069	0.51	(0.49 - 0.52)	49.3	(47.6 - 51.0)
	2 - 7 weeks	148 979	48581	0.63	(0.62 - 0.64)	36.8	(35.7 - 37.8)
	8 - 13 weeks	161 049	44350	0.81	(0.79 - 0.82)	19.5	(18.0 - 20.9)
	14 - 19 weeks	158 457	12879	1.01	(0.99 - 1.04)	-1.2	(-3.6 - 1.3)
	20 - 25 weeks	156 600	11622	1.13	(1.10 - 1.16)	-13.1	(-16.4 - -9.9)
	26 - 31 weeks	153 908	15476	1.22	(1.18 - 1.25)	-21.7	(-25.2 - -18.3)
	32 - 37 weeks	150 886	8341	1.21	(1.17 - 1.25)	-21.0	(-25.1 - -17.0)
	38 - 43 weeks	147 626	8092	1.07	(1.04 - 1.11)	-7.3	(-11.1 - -3.6)
	44 - 49 weeks	135 592	12394	1.06	(1.03 - 1.10)	-6.3	(-9.9 - -2.8)
	≥50 weeks	59 493	6394	1.23	(1.18 - 1.28)	-23.3	(-28.3 - -18.4)

*Result not shown for strata with <5 events. *Adjusted for age, sex, region of residence, country of birth and risk group. ¤ Vaccine effectiveness= (1-aHR)*100*

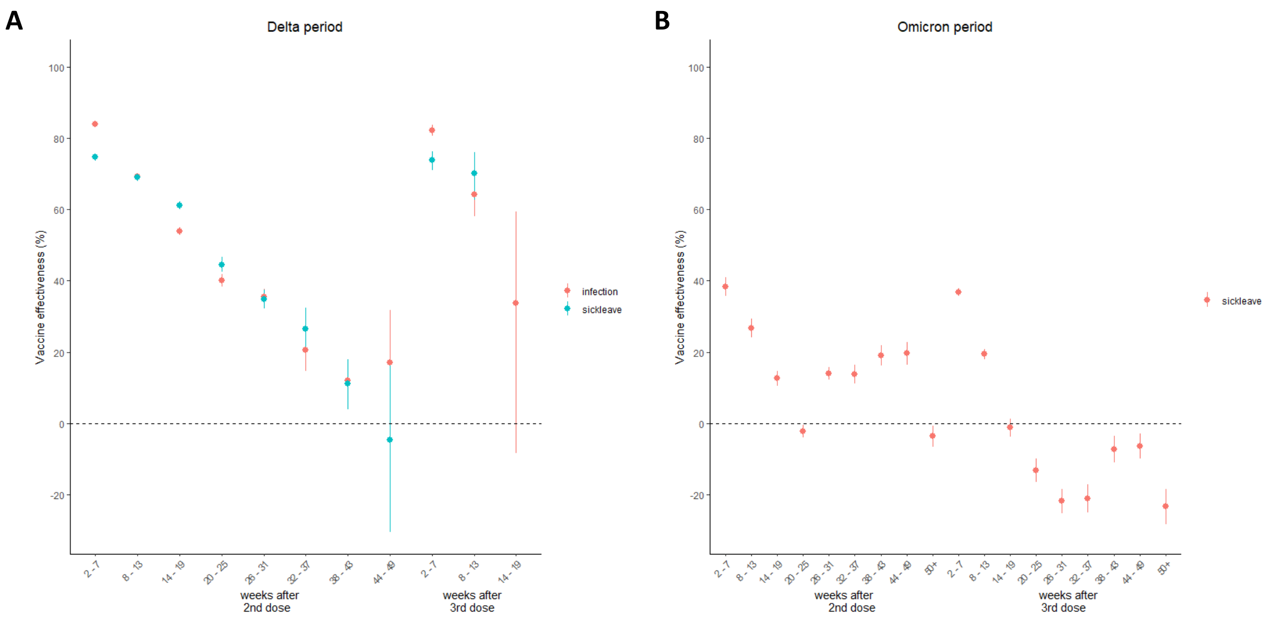

Figure S1. *Estimated adjusted vaccine effectiveness for COVID-19 vaccination status against infection and COVID-19 sick leave during the period of Delta (A) and Omicron dominance (B) among employed individuals (24-65 years) in Norway.*

References

1. Norwegian Institute of Public Health. Emergency preparedness register for COVID-19 (Beredt C19). Oslo: Norwegian Institute of Public Health; 2021 [cited 2022 05 Nov]. Available from: https://www.fhi.no/en/id/infectious-diseases/coronavirus/emergency-preparedness-register-for-covid-19/.
2. Norwegian Institute of Public Health. Påvisning og overvåkning av SARS-CoV-2-virusvarianter. Oslo: Norwegian Institute of Public Health; 2021 [cited 2022 05 Nov]. Available from: https://www.fhi.no/nettpub/coronavirus/testing/pavisning-og-overvakning-av-sars-cov-2-virusvarianter/.
3. Indseth T, Godøy A, Kjøllesdal M, Arnesen T, Carelo C, Vinjerui KH, et al. Covid-19 by country March 2020-February 2021. 2021.
